# Disorder perception is the adaptive interpretation of social cues, not just a sensitivity to randomness

**DOI:** 10.3389/fpsyg.2015.00124

**Published:** 2015-02-10

**Authors:** Daniel T. O'Brien

**Affiliations:** ^1^School of Public Policy and Urban Affairs, School of Criminology and Criminal Justice, Northeastern UniversityBoston, MA, USA; ^2^Boston Area Research Initiative, Harvard UniversityCambridge, MA, USA

**Keywords:** broken windows theory, community perception, urban neighborhoods, physical disorder, social disorder

This past June Kotabe (2014) presented the World is Random (WIR) model for disorder perception. He argued that it can explain phenomena across domains and multiple levels of analysis, most importantly the dynamics articulated by the popular sociological theory of “broken windows” (BWT). There are two critical problems with this claim. First, Kotabe makes a false dichotomy between “psychological” mechanisms and “social” behavior, obscuring any social function that disorder perception might have. Second, the author gave only a cursory summary of BWT and the behavioral patterns it predicts, meaning WIR has been evaluated not against the actual theory fit to an anecdotal sampling of findings associated with it. Notably, these two issues are each dealt with by an existing psychological theory called community perception that the author does not address. This perspective states that the perception and interpretation of disorder in neighborhoods is part of an evolved adaptation for evaluating the social character of collectively inhabited spaces (O'Brien and Wilson, 2011).

The WIR model posits that “perceiving disorder primes concepts related with randomness… [leading] us to (accurately) believe we have less control over outcomes … and to (erroneously) believe we have less control over ourselves,” which in turn “may have a variety of affective, judgmental, and behavioral consequences” (Kotabe, 2014, pg. 2). This indeed imagines disorder perception as a cognitive mechanism independent of social processes, the author's stated goal. However, it is not obvious that this is wise if one seeks to explain the diverse behavioral effects of disorder and the sociological dynamics that follow. The core problem is the insistence on distinguishing “psychological” from “social” mechanisms. Humans are social beings and have many psychological mechanisms whose primary task is to navigate social contexts. Attempting to separate the cognitive from the social in these cases would be to lose sight of the functions that they serve.

The question then is whether the affective, judgmental, and behavioral consequences of disorder perception are more consistent with that of a socially-oriented mechanism, or WIR's “within-one-head” model. To probe this question I turn first to BWT itself. In their seminal essay, Wilson and Kelling (1982) argued that disorder in public spaces—graffiti, drunkenness—signals that a community is unable to enforce local social norms, making it a potential safe haven for crime. In turn this signal would encourage behavior leading to the escalation of disorder and crime through two pathways. The more famous is that the sense of impunity would motivate those with delinquent inclinations to act on them. The second is that average citizens would feel threatened, becoming socially withdrawn. The consequent erosion to the community's social dynamics would further weaken the ability to enforce norms.

WIR accounts for some aspects of each of these two behavioral responses by citing disorder's tendency to impart a sense of diminished control: would-be transgressors become more impulsive, and upstanding citizens feel threatened by the unpredictability of the environment. Another psychological model called community perception, proposed by O'Brien and Wilson (2011), accounts for these same patterns of response through a cognitive mechanism that is socially oriented. We argued that a group-living species must navigate social environments and quickly infer the kinds of encounters that might occur there. Thus, humans would be expected to have a psychological adaptation that heuristically interprets information regarding local social dynamics, and then translates that information into behavior. In a series of studies they found that naïve individuals were able to generate accurate assessments of the social quality of unfamiliar neighborhoods using only photos of the physical context (agreement with resident surveys, *R*^2^ ≈ 0.50), judgments based almost entirely on the presence of disorder (*R*^2^ ≈ 0.90). Participants were also less likely to cooperate in a prisoner's dilemma when paired with a person from a neighborhood with higher levels of disorder, indicating an adaptive social response to a potentially threatening context.

In order to compare these two models, let us first turn to the response of the would-be transgressor. The WIR model posits that the perception of disorder instills a lack of self-control, thereby releasing the individual of any inhibitions surrounding uncivil and criminal behavior. Though, as Kotabe (2014) notes, most existing experimental evidence is somewhat ambiguous on this point, a study by Brown and Bentley (1993) found that burglars are more likely to target houses that have evidence of disorder, owing to the perceived lower likelihood of being apprehended. Far from a loss of self-control, these burglars, in keeping with the community perception model, are using social information from cues of disorder to take adaptive action.

The second pathway of BWT rests on disorder's ability to induce fear of crime. As mentioned, WIR posits that this is part of a more generalized sense of fear arising from the threats of an unpredictable environment. This is then projected upon the social environment. In contrast, community perception would argue that individuals are actively searching the environment for social information that might indicate the local crime level. Two aspects of this process support the latter view over the former. First, people's definitions of disorder are neither universal nor idiosyncratic, but are socialized to the norms of their local community. O'Brien et al. (2014) found that when viewing neighborhoods from another city, participants were less accurate. Importantly, these inaccuracies arose because participants were focusing on cues that were informative in their home city, but not in the one they were viewing. Second, there is mounting evidence that disorder is just one of multiple cues that people use to evaluate the safety of a neighborhood. A number of studies have shown that lawn decorations and other signs of personalization have an effect opposite to that of disorder (Harris and Brown, 1996; O'Brien and Wilson, 2011; O'Brien et al., 2014). Similarly, preliminary work has found that neighborhood investment in the form of building projects lowers people's perception of incivilities in the neighborhood (Montgomery and O'Brien, 2014). Last, in their paper on “the social construction of broken windows,” Sampson and Raudenbush (2004) found that residents of Chicago also used the proportion of Black residents in a neighborhood to estimate the local crime rate.

All of this would suggest that the sociological dynamics described by BWT are best explained by a social adaptation that uses heuristic cues, including but not limited to disorder, to generate locally adaptive behavior. In turn, the claims made by Kotabe (2014) are not commensurate with the WIR model's limitations. It is possible that humans have a primitive, non-social sensitivity to disorder that can influence affect and behavior. This may have even been a pre-adaptation for community perception, as it would be easier to evolve a social aversion to cues that are already aesthetically unsettling. That said, the WIR model cannot on its own explain a process that is inherently social.

## Conflict of interest statement

The author declares that the research was conducted in the absence of any commercial or financial relationships that could be construed as a potential conflict of interest.
